# The Association between Parenting Behavior and Executive Functioning in Children and Young Adolescents

**DOI:** 10.3389/fpsyg.2017.00472

**Published:** 2017-03-30

**Authors:** Zrinka Sosic-Vasic, Julia Kröner, Sibylle Schneider, Nenad Vasic, Manfred Spitzer, Judith Streb

**Affiliations:** ^1^Department of Psychiatry and Psychotherapy III, University Clinic of UlmUlm, Germany; ^2^Department of Sociology, University of AugsburgAugsburg, Germany; ^3^Department of Psychiatry and Psychotherapy, Clinical Centre ChristophsbadGöppingen, Germany; ^4^Transfercenter of Neuroscience and Learning, University of UlmUlm, Germany; ^5^Department of Forensic Psychiatry and Psychotherapy, University of UlmUlm, Germany

**Keywords:** executive functions, self-regulation, cognitive development, parenting, school, children, adolescents

## Abstract

Executive functioning (EF) is associated with various aspects of school achievement and cognitive development in children and adolescents. There has been substantial research investigating associations between EF and other factors in young children, such as support processes and parenting, but less research has been conducted about external factors relating to EF in older children and adolescents. Therefore, the present study investigates one possible factor that could correlate with EF in school-age children and adolescents: parenting behavior. The cross-sectional study design gathered data from 169 children in primary schools, middle-schools, and Gymnasien, and their corresponding parents. All children underwent a standardized task to measure EF, the computer-based Erikson Flanker task, which evaluates EF as a function of error rates and response time. A self-report questionnaire was used to assess parenting behavior. Multilevel analysis was implemented to test the effects of parenting behavior on EF in school-age children. The results show significant associations between various parenting behaviors and children's EF: High scores on parental involvement or parental responsibility are associated with low error rates on the Erikson Flanker task, whereas high parental scores on inconsistent discipline are associated with high error rates. These correlations between parenting behavior and EF remained significant despite controlling for child age, maternal education, family income, and baseline performance (i.e., congruent trials on the Erikson Flanker task). No associations were found between parental behavior and reaction time on the Erikson Flanker task. These results indicate the important association between parenting behaviors and EF skills in school-age children, and foster the necessity to inform parents about ways in which they can optimally support their children's cognitive development.

## Introduction

Executive functions (EF), also called cognitive control skills, are part of self-regulatory mechanisms that include various cognitive processes of higher order that are involved in goal-directed behavior (Luria, 1966, 1976; Vygotsky, 1980; Stuss and Benson, 1986), such as attention-shifting, problem solving, planning, working memory, and inhibition (Pennington and Ozonoff, 1996; Stuss and Levine, 2002; Fuster, 2008; Garon et al., 2008; Diamond, 2013). While some of these behaviors are apparent constructs of what we call intelligence (for a detailed definition see Duggan and Garcia-Barrera, 2015), there is still inconsistent evidence for the relation of EF and the latter (Arffa, 2007). For example, it has been demonstrated that working memory processes have a strong correlation with psychometric measures of intelligence, whereas inhibition and attention-shifting displayed weak correlations with intelligence (Friedman et al., 2006). However, a significant amount of research has demonstrated that several aspects of EF and self-regulation abilities are strongly correlated with academic achievement (e.g., Blair and Razza, 2007; Duncan et al., 2007; Li-Grining et al., 2010; Fuhs et al., 2014). It has been hypothesized that higher EF skills allow children to meet classroom demands quicker—i.e., those children have better attention capacities, memory of classroom rules, and are more capable to engage in academic content—which may enable them to benefit more from the academic environment that they are in Fuhs et al. (2015) demonstrated that teachers' ratings as well as measurements of EF were directly correlated with academic achievement in prekindergarten children. Another study by Blair and Razza (2007) showed that teachers' ratings of effortful control and measurements of these EF skills were directly associated with children's literacy and mathematics skills during kindergarten. Studies measuring EF in elementary and early middle-school aged children found that children's EF skills before and during elementary school predicted their competence in sixth grade (Jacobson et al., 2011). Furthermore, the same study showed that teachers reported higher academic and behavioral difficulties in students with weaker EF skills.

It is interesting that despite the scientific finding, which indicates that EF skills are highly heritable—with up to 99% of EF components being due to genetics (Friedman et al., 2008)—EF skills mainly develop during childhood and adolescence as a function of prefrontal cortical maturation, as the prefrontal cortex (PFC) establishes its reciprocal connections over the lifespan developmental course by connecting to subcortical brain structures and other cortical regions (e.g., Fuster, 1997, 2002; Diamond, 2002; Heyder et al., 2004). Therefore, it has been hypothesized that relational experiences, such as social interactions, may be correlated with children's and young adult's neurocognitive development and EF (e.g., Nelson and Bloom, 1997; Hickman et al., 2000; Carpendale and Lewis, 2006; Lewis and Carpendale, 2009). Support for this type of social relational approach stems from several studies. For example, Sosic-Vasic et al. (2015) analyzed school-aged children between 8 and 14 and demonstrated that EF skills were modulated by teacher's autonomy support in classroom. In addition, teacher's supportive behavior was linked not only to child EF but also to child motivation. Thus, teachers that support autonomy in learning situations are related to children that perform better on executive function tasks.

In further support for a social relational approach to EF development, specifically the quality of parenting behaviors proved as an important modulating candidate (Bernier et al., 2010). Among these, sensitive parenting (e.g., Towe-Goodman et al., 2014; Sulik et al., 2015) and parental scaffolding (e.g., Lowe et al., 2014) showed to be associated with EF in young children in several studies. It is assumed that parents serve as self-regulating catalysts and attention-switches for their children while their brain structures form, which will later be in charge of these mechanisms (e.g., Carlson, 2009). Children and parents establish a strong bond during early and middle childhood—i.e., the sensitive period during which EF continues to develop. For example, Bernier et al. (2015) demonstrated that securely attached toddlers performed better on EF tasks at school entry than their insecurely attached peers, and further had less EF problems as reported by their teachers. Moreover, the level of maternal sensitivity, autonomy support, and mind-mindedness have proven to be related to EF, with autonomy support being the strongest predictor of EF in babies and toddlers (Bernier et al., 2010). Maternal autonomy support during the first 3 years of life also predicted enhanced EF functions during kindergarten, and greater academic achievement in elementary and high-school children (Bindman et al., 2015). It has also been previously documented that parent-child relationships—i.e., relationships that are based on positive parenting behavior (Fatima et al., 2016)—are related to children's cognitive development and EF, specifically within the United States (Portes et al., 2000). The same study also showed that parental associations with EF might be culturally sensitive, due to cultural differences in parenting behaviors and family environments. However, parent-child relationships have also been associated with EF skills in Asian adolescents (Fatima et al., 2016). Their study specifically showed that perceived neglect was negatively correlated with measures of EF. Taking a closer look at parental care and EF in infants, toddlers, and preschool children, it can be assumed that there might be differences between father's and mother's sensitive and supportive parenting which, in turn, is correlated with EF (Towe-Goodman et al., 2014; Lucassen et al., 2015).

Thus, while there is substantial evidence that EF abilities—which lie within the normal range—are important mechanisms related to pre-school children's social and cognitive functioning, there is still a lack of research considering associations of EF amongst school-aged children. The current study shall account for this gap in research and reveal further insight on the associations between parenting and EF in school-age children.

In summary, emerging research has highlighted the importance of parental care, which is needed to provide the necessary support and opportunities in order for EF skills to emerge (Carlson, 2009; Bernier et al., 2012). However, it is interesting that despite the evidence for enormous brain plasticity in children and a plethora of research demonstrating the effects of early environmental experiences on brain development, specifically within the context of parental caregiving and EF abilities, there is still little knowledge about associations between parenting behavior and EF skills in school-aged children between the ages 9 and 14. The PFC has an extended period of development (De Bellis, 2001; Gunnar et al., 2006) and due to the PFC's correlation with the development of EF skills, there are substantial changes in EF functioning across and beyond childhood (for reviews see Diamond, 2006; Garon et al., 2008), with some aspects of EF skills developing far beyond adolescence (Carriedo et al., 2016). It has also been previously demonstrated that parent-child relationships and their correlations with EF in children are culturally sensitive (Portes et al., 2000), however, to the best of our knowledge, there has been no study investigating the associations between parenting behaviors and school-age children's EF skills within a European context.

In order to account for this gap in knowledge, we investigated the associations between seven dimensions of parenting—i.e., positive involvement, supervision, and monitoring, positive discipline, consistency with discipline, use of corporal punishment, responsible parenting, and authoritarian parenting—and EF skills in children and adolescents between 9 and 14. Understanding possible associations between external factors—such as parenting behavior—and EF functions is crucial, because EF is correlated with academic achievement and various positive social behaviors, which together may pave the way for later success in life.

The present study implements a cross-sectional design, taken from a representative sample of primary and junior high-school children. We administered a well-established test to extensively measure EF skills, and administered a self-report questionnaire to the respective parents in order to gather information about their parenting behavior. Based on previous literature, we expect that positive parenting behaviors such as positive involvement, positive discipline, and consistency with discipline will correlate with higher EF skills; while we expect a moderate amount of supervision and monitoring, and a low level of corporal punishment to be positively associated with higher EF skills.

## Methods

### Participants

In a cross-sectional study we tested 169 children and questioned their parents. We investigated 35 primary school children (grades: 3 and 4, mean age = 9.68, *SD* = 0.59, range = 9–11 years, 15 boys/20 girls), 89 junior high school children attending so called Middle schools (grades: 5 and 6, mean age = 11.95, *SD* = 0.82, range = 11–14 years, 41 boys/48 girls), and 43 junior high school children attending so called Gymnasien (grades: 5 and 6, mean age = 11.61, *SD* = 0.66, range = 11–13 years, 20 boys/23 girls). The parents' questionnaire was completed by the primary caregiver, i.e., the mother (71%), the father (4%), both (23%) or others (2%, e.g., grandmother or legal guardian). Children and their parents volunteered for participation after an informative meeting. Children and parental informed consent to participate were obtained in writing prior to data collection. The study was approved by the Internal Review Board of the Medical Faculty of the University of Ulm.

### Procedure

Children attended regular school hours and were tested during those times, in order for them to not have to appear at additional time. In order to carry out tests on executive functioning skills, children were placed in a computer room and were tested in small groups. Parent questionnaires were distributed to the children at school and delivered to the parents through their respective children. Parents or designated caregivers answered the questionnaire at home and handed it back after a few days.

### Material

#### Executive function tests

**Figure 1:**
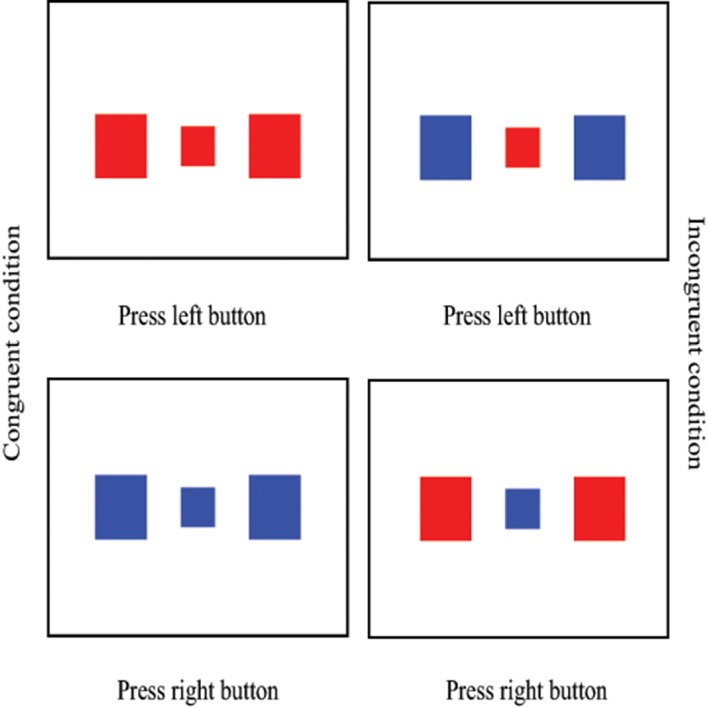
**Examples of the different trials of the Eriksen Flanker Task**. This figure was adapted from Sosic-Vasic et al. (2015).

To assess executive functions in children we implemented the Eriksen Flanker Task (Eriksen and Eriksen, 1974) as computer based test (see Figure 1). The task is appropriate for the ages 4 to adults.

During the Eriksen Flanker Task children focused on the color of a small red or blue rectangle (i.e., the target) presented in the center of the screen. The target stimulus was flanked by two rectangles that appeared 4.5 cm to the left and to the right of the target and were either red or blue. Height and width of each flanker was three times that of the target. Target and flanker were displayed simultaneously. In the congruent condition, both, target and flanker, matched in color. In the incongruent condition the flankers were blue when the target was red and vice versa. Children were instructed to respond depending on the color of the target by pressing the left or right mouse button with their dominant hand. Stimuli were presented until the button was pressed, with the inter stimulus interval being 500 ms. Appearance of the four combinations of target and flanker color was equiprobable (blue-blue, blue-red, red-blue, red-red). Prior to the test, children completed 60 practice trials (half congruent, half incongruent) and the final test comprised 40 congruent and 40 incongruent trials, with randomized order of trials.

The task was conducted via computer presentation positioned at eye level, at a distance of 50 cm from the children. The mouse was placed on a table in front of the children at a comfortable distance. The response keys were the left and right mouse button marked with different stickers. In general, children were instructed to respond as quickly and accurately as possible.

The task assesses all three major executive function domains—inhibition, working memory, and cognitive flexibility (see Diamond, 2013). Both the congruent and incongruent conditions require memory of the rules. The incongruent condition also requires inhibition and cognitive flexibility to change the focus of attention and stimulus-response mappings.

The output of the executive function test was prepared as follows: Response times faster than 200 ms were considered too fast to be in response to the stimulus and excluded from further analyses of error rate (accuracy) and reaction time (speed). The error rate was calculated by dividing the number of incorrect responses by the sum of correct plus incorrect responses. The reaction time was calculated for correct responses only. The dependent measures mean reaction times and mean error rate were computed separately for each participant and each condition.

#### Parent questionnaire

Parents completed the German version of the Alabama Parenting Questionnaire (Reichle and Franiek, 2009), which was created by translating, adapting and extending the original questionnaire by Frick (1991) to measure the dimensions of positive parenting behavior (example item: You let your child know, when he/she is doing a good job with something.), involvement (example item: You have a friendly talk with your child.), poor monitoring (example item: Your child fails to leave a note or to let you know where he/she is going.), inconsistent discipline (example item: You threaten to punish your child and then do not actually punish him/her.), and corporal punishment (example item: You spank your child with your hand when he/she has done something wrong). Reichle and Franiek (2009) appended two additional scales to assess authoritarian parenting (example item: If your child wants you to make an exception, you insist on your rules, so it is clear who has the say in the family) and responsible parenting (example item: You discuss with your child what he/she can do in his/her free time). The German version of the questionnaire consists of 40 items, which are rated on a 5-point Likert scale with anchors of never (1) and always (5). The psychometric properties were good to satisfactory, with half year retest-reliabilities ranging from *r* = 0.57 to *r* = 0.77.

### Statistical analyses

Question 1: Do the executive function tests work?

The executive function test was analyzed by within-subjects ANOVAs in order to verify that the student's performances were systematically influenced by the different task conditions. As dependent variables we considered both reaction times and error rate. The independent variable is the task condition (congruent and incongruent).

Question 2: How does parenting relate to their children's executive functions?

Specific associations with the dependent variables were analyzed by linear mixed models. Considering that the present study was conducted in a school setting, where students are nested in schools, we used a multilevel modeling approach—the SPSS mixed procedure (SPSS, 2005). The school level was included as random effect to account for common variance. Random-intercept models were estimated in which the intercepts were allowed to vary randomly but with fixed effects for all predictor variables. The method of estimation was restricted maximum likelihood. Error rate and reaction time of the Eriksen flanker task were tested by introducing the incongruent trials as dependent and the congruent trials as independent variable (=fixed effect). The difference between them displays the additional allocation of executive function capacities while coping with the more challenging task. The analyses were computed separately for error rate and reaction time. The scales of the German version of the Alabama parenting questionnaire and—to account for possible bias—child age, maternal education, and family income were included as fixed effects.

## Results

Table 1 shows the descriptive statistics for the Eriksen flanker task and the scales of the German version of the Alabama parenting questionnaire.

**Table 1:** **Descriptive statistics**.

	***M***	***SD***	**Minimum**	**Maximum**
**ERIKSEN FLANKER TASK**				
Reaction time: congruent	585.98	90.20	364.33	874.00
Reaction time: incongruent	619.76	135.18	331.50	1526.00
Error rate: congruent	0.09	0.17	0.00	0.94
Error rate: incongruent	0.11	0.18	0.00	0.98
**ALABAMA PARENTING QUESTIONNAIRE (German Version)**				
Involvement	2.71	0.60	0.50	4.00
Positive parenting	3.24	0.54	1.33	4.00
Poor monitoring	0.36	0.47	0.00	2.67
Inconsistent discipline	1.33	0.69	0.00	2.83
Corporale punishment	0.38	0.52	0.00	2.50
Responsible parenting	0.33	4.00	2.59	0.72
Authoritarian parenting	0.33	4.00	2.40	0.67

In the Eriksen Flanker Task, reaction time was faster and error rate was lower in the congruent-, than in the incongruent-condition. The descriptive statistics for the parenting questionnaire disclose that parents have high scores in the domains positive parenting and involvement but low scores in authoritarian and responsible parenting.

Table 2 displays the correlations among the six dimensions of parenting behavior. As can be seen there are strong correlations between positive parenting and almost all other subscales, whereas authoritarian parenting seems to be uncorrelated to all other dimensions.

**Table 2:** **Correlations among dimensions of parenting behavior**.

	**Responsible parenting**	**Authoritarian parenting**	**Inconsistent discipline**	**Involvment**	**Corporale punishment**	**Poor monitoring**
Positive parenting	0.307^**^	0.104	−0.235^**^	0.370^**^	−0.336^**^	−0.378^**^
Responsible parenting		0.238^**^	0.090	0.156^*^	−0.118	−0.166^*^
Authoritarian parenting			0.111	0.050	0.050	−0.039
Inconsistent discipline				−0.056	0.195^*^	0.349^**^
Involvment					−0.272^**^	−0.303^**^
Corporale punishment						0.383^**^

** p < 0.01;

* *p < 0.05*.

Question 1: Do the executive function tests work?

The within-subject ANOVAs of the Eriksen flanker task disclose a significant difference between the incongruent and congruent trials with regard to reaction time [*F*_(1, 168)_ = 46.94; *p* < 0.001; part. Eta^2^ = 0.218] as well as with regard to error rate [*F*_(1, 168)_ = 7.42; *p* < 0.01; part. Eta^2^ = 0.043].

This result confirms that the student's responses were systematically elicited by the task conditions. Further analyses take baseline performance (=congruent condition) into account and display the additional allocation of executive functions while coping with the more demanding, incongruent condition.

Question 2: How does parenting relate to children's executive functions?

The effects of parenting on student's executive functions were examined using linear mixed models.

The results can be seen in Table 3, separately for error rate (left part) and reaction time (right part). Children's executive function is associated with their parent's support. Children, whose parents rate themselves as highly responsible show significant lower error rates, whereas children, whose parents assess themselves as being highly inconsistent, disclose significant higher error rates. Maternal education and family income were not related to children's executive function.

**Table 3:** **Results [***b*** = estimates of fixed effects; ***SE***(***b***) = standard errors] of linear mixed model analyses predicting error rate and reaction time for incongruent trials of the Eriksen flanker task**.

**Predictors**	**Eriksen flanker task (incongruent trials)**			
	**Error rate**		**Reaction time**	
	***b***	***SE*(*b*)**	***b***	***SE*(*b*)**
Congruent trials	0.95^**^	0.04	1.02^**^	0.07
Child age	−0.04^*^	0.02	−31.7	14.18
**MATERNAL EDUCATION (Reference = no/basic school qualification)**				
Secondary	0.02	0.04	27.18	34.32
University	0.04	0.05	24.71	40.28
**FAMILY INCOME (Reference = lower than 1,750 Euro)**				
1,750 through 3,000 Euro	0.03	0.03	−3.58	22.24
higher than 3,000 Euro	−0.02	0.04	9.93	29.91
**ALABAMA PARENTING QUESTIONNAIRE (German Version)**				
Involvement	−0.04	0.02	−4.41	18.91
Positive parenting	0.003	0.03	16.34	22.22
Poor monitoring	0.03	0.04	3.57	27.26
Inconsistent discipline	0.04^*^	0.02	20.37	15.91
Corporal punishment	−0.04	0.03	3.75	25.51
Responsible parenting	−0.05^*^	0.02	−4.98	14.99
Authoritarian parenting	0.01	0.02	−2.44	15.22
*R*^2^	0.47		0.22	

** p < 0.01;

* *p < 0.05; R^2^ = Level-1 reduction in variance estimates; All continuous predictors were centered within cluster (schools) before being included in the model*.

Table 3 displays further data about the reduction in variance estimate (*R*^2^) for the within-school portions of the model. The total amount of fixed effects accounted for 47 percent of the within-school variability in student's error rate. The reported *R*^2^ result is quite high, indicating that the data is a good fit to the regression model.

## Discussion

Building onto previous studies, which demonstrated that parenting might play an important role in the development of EF in infants and small children (e.g., Bernier et al., 2010; Blair et al., 2011; Hammond et al., 2012), we implemented a multilevel-hierarchical analytical model to test various ways in which seven aspects of the quality of parental care—involvement, positive parenting, poor monitoring, inconsistent discipline, corporal punishment, responsible parenting, and authoritarian parenting—might be associated with the development of EF in school-age children between the ages 9 and 14, to account for a gap in research within this specific population.

The analysis showed that inconsistent discipline, and responsible parenting is associated with executive functioning skills in school-age children. First, parent's inconsistent discipline was associated with the children's error rates, in that children who have higher error rates, have parents who are more inconsistent in disciplining their children. Going into more detail about the aforementioned parenting styles, which are authoritative, authoritarian, permissive, and neglectful (e.g., Baumrind, 1971), these results could be an indicator of a generally less favorable parenting style, such as permissive or neglectful. Children of parents who are nested within one of those parenting styles, usually demonstrate a plethora of negative developmental outcomes, such as less advanced cognitive development, when compared to children of parents who apply the favored authoritative parenting style (e.g., Dekovicé and Janssens, 1992; Crockenberg et al., 1996; Paterson and Sanson, 1999). Furthermore, executive functioning tasks are built around the requirement to flexibly adapt to errors when they occur. In order to improve the error rate in upcoming tasks, the child has to be able to learn from their previous mistakes. Children of parents who demonstrate inconsistent discipline practices might not be as equipped to learn from previous mistakes, because they do not experience consistent negative reinforcement upon unfavorable behavior. This behavioral drawback might then lead to a delay in the development of autonomous behavior, which is necessary for self-regulation and functioning in the social and academic world. Within this context it is important to note that previous research suggests that autonomy can promote self-regulation, which in turn enhances the capacity to be open to failures (Hodgins and Liebeskind, 2003; Weinstein et al., 2011). Therefore, there appears to be a direct link between parental inconsistent discipline practices and the development and improvement of EF skills.

Second, responsible parenting was associated with error rate in the Erikson Flank Task. The results revealed that highly responsible parents have children who have lower error rates than children with parents who demonstrate lower levels of responsible parenting. Responsible parenting within this context is defined as behavior that is intended to maintain the child's integrity by providing constructive, non-impulsive, and emotionally controlled actions and comments in the areas of caretaking, grooming, safety, and parenting; with the intend to do no harm to the child and teaching the child to not harm others. This form of parenting is guiding the child to behave in conformity with normative social standards, while at the same time providing the child with freedom of action for them to choose, knowing the future consequences of their behaviors and actions (Reichle and Franieck, 2009). This result is aligning with previous research that suggests the importance of parenting in the development of EF skills (e.g., Blair et al., 2014). The same study showed that parental sensitivity and responsiveness correlate with child EF at a later age. Both of these components are inherent in the construct of responsible parenting: Responsible parents are teaching their children social norms and guide them through their lives by letting them make their own decisions under their supervision, which provides freedom of action from which the child can explore future behavioral outcomes. However, most of the previous studies only focused on young children (e.g., Blair et al., 2014, Bindman et al., 2015). Therefore, the presented research extends onto previous knowledge, by providing important insight into parenting behavior and EF outcomes in older children and young adolescents. It becomes apparent that sense of responsibility and consistency in discipline are not only important within the developmental context of EF skills in young and middle-aged children, but also for pre-teens and adolescents. Knowing that the PFC evolves far into adolescence, and EF skills being directly contingent upon this development, it appears that parental behavior is directly linked to EF outcomes in school-age children.

## Limitations

The present study comprises of cross-sectional data, collected at a single measurement point, and therefore does not claim to be able to make future predictions of parental and child behaviors. The capacity of this data to draw causal relations between the investigated variables is therefore also limited and warrants further research and exploration. Furthermore, there has been no measurement of general intelligence. This might be important for mediation analysis in future research, specifically within the German school context, as children attending the Gymnasium—on average—score higher on so-called IQ tests than children from middle-schools. Moreover, the present study did not evaluate the associations between parenting behavior and the various components of EF separately. Therefore, it might be important for future research to investigate whether parenting styles predict different components of EF to different extends. The present study also assessed parenting behavior with a self-report questionnaire. Therefore, parents could have responded to the questions in a socially desirably way. Future research could test for this fallacy by implementing objective measures of parenting behavior. Another component that seems to be crucial to EF is the genetic heritage of EF skills. Hence, it might be possible that parents with poor EF skills genetically pass them on to their children. It is also possible, that there is an interactional effect between parenting behavior and genetic heritage, such as parents with poor EF skills also have less optimal parenting styles. These hypotheses also warrant further scientific investigation. Other possibly important variables, such as temperamental factors or class level have also not been taken into consideration. The evaluated population only consisted of school-age children in Saxony, a state in Eastern Germany. Hence, the data generalizability is limited and necessitates further elaboration across other German states, countries, and nations.

## Ethics statement

This study was carried out in accordance with the recommendations of the Internal Review Board of the Medical Faculty of the University of Ulm with written informed consent from all subjects and their legal representatives. All subjects gave written informed consent in accordance with the Declaration of Helsinki. The protocol was approved by the Internal Review Board of the Medical Faculty of the University of Ulm.

## Author contributions

ZS and JS developed the study idea as well as the study design. Both parties were furthermore involved in sample recruitment, data analysis, and contributed to manuscript writing and editing. JK Contributed to the data analysis, the manuscript writing and editing, and conducted the literature research. SS helped developing the study design, and participated in data analysis and manuscript editing. NV assisted in developing the study design, analyzing the data, and manuscript editing. MS helped developing the study idea and edited the manuscript.

### Conflict of interest statement

The authors declare that the research was conducted in the absence of any commercial or financial relationships that could be construed as a potential conflict of interest.
